# Susceptibility of different mouse strains to oxaliplatin peripheral neurotoxicity: Phenotypic and genotypic insights

**DOI:** 10.1371/journal.pone.0186250

**Published:** 2017-10-11

**Authors:** Paola Marmiroli, Beatrice Riva, Eleonora Pozzi, Elisa Ballarini, Dmitry Lim, Alessia Chiorazzi, Cristina Meregalli, Carla Distasi, Cynthia L. Renn, Sara Semperboni, Lavinia Morosi, Federico A. Ruffinatti, Massimo Zucchetti, Susan G. Dorsey, Guido Cavaletti, Armando Genazzani, Valentina A. Carozzi

**Affiliations:** 1 Experimental Neurology Unit, School of Medicine and Surgery, University of Milano-Bicocca, Monza, Italy; 2 Department of Pharmaceutical Sciences, University of Piemonte Orientale, Novara, Italy; 3 School of Nursing, Department of Pain and Translational Symptom Science, University of Maryland, Baltimore, Maryland, United States of America; 4 Department of Oncology, IRCCS Istituto di Ricerche Farmacologiche Mario Negri, Milan, Italy; University College London, UNITED KINGDOM

## Abstract

Peripheral neurotoxicity is one of the most distressing side effects of oxaliplatin therapy for cancer. Indeed, most patients that received oxaliplatin experience acute and/or chronic severe sensory peripheral neuropathy. However, despite similar co-morbidities, cancer stage, demographics and treatment schedule, patients develop oxaliplatin-induced peripheral neurotoxicity with remarkably different severity. This suggests individual genetic variability, which might be used to glean the mechanistic insights into oxaliplatin neurotoxicity. We characterized the susceptibility of different mice strains to oxaliplatin neurotoxicity investigating the phenotypic features of neuropathy and gene expression profiles in dorsal root ganglia of six genetically different mice strains (Balb-c, C57BL6, DBA/2J, AJ, FVB and CD1) exposed to the same oxaliplatin schedule. Differential gene expression in dorsal root ganglia from each mice strain were assayed using a genome-wide expression analysis and selected genes were validated by RT-PCR analysis. The demonstration of consistent differences in the phenotypic response to oxaliplatin across different strains is interesting to allow the selection of the appropriate strain based on the pre-defined read-out parameters. Further investigation of the correlation between gene expression changes and oxaliplatin-induced neurotoxicity phenotype in each strain will be useful to deeper investigate the molecular mechanisms of oxaliplatin neurotoxicity.

## Introduction

Oxaliplatin is a 3^rd^ generation compound of the platinum drug family effectively employed for the treatment of colorectal cancer, the third leading cause of death in western countries. Even if earlier diagnosis and aggressive cancer treatment allow a longer life expectancy, long-term treatment-related side effects can severely compromise patients’ quality of life after oxaliplatin treatment. Since oxaliplatin preferentially damages the peripheral sensory fibers and dorsal root ganglia (DRG) neurons [1], which lack the protection of the blood brain barrier, one of the most debilitating side effects of oxaliplatin-based regimens is peripheral neurotoxicity. Oxaliplatin treatment produces an acute and transient neuropathy (rate up to 90%) with neuropathic pain elicited by exposure to cold and a chronic sensory syndrome (rate 40–80%) with similar, but enduring characteristics complicated by nerve functional impairment and structural degeneration [2–7]. The exact mechanisms underlying oxaliplatin-induced neurotoxicity (OIPN) remain poorly understood. Alterations in ion channels and calcium signalling, oxidative stress, mitochondrial failure and drug membrane transporters are only some of the most studied mechanisms of OIPN [8, 9]. Despite similar co-morbidities, demographic profile, cancer stage and treatment schedule, oxaliplatin-treated patients can develop OIPN with different severity [10]. Individual variability in drug toxicity response has suggested that specific genetic variants determining over-expression or down-regulation of genes involved in drug disposition, metabolism, detoxification, channels functioning and DNA repair could be associated with OIPN susceptibility and severity [11–16], but this still remains a matter of scientific debate [5, 17, 18].

This variability can also be observed in animal models of chemotherapy-induced peripheral neurotoxicity, where genetic variations between mice strains can influence response to drug treatment and determine different toxicity phenotypes [19, 20].

In this study, we tested the different susceptibility to oxaliplatin neurotoxicity through a phenotypic characterization of OIPN in several inbred and one outbred strains of mice. Inbred strains, generated by sib mating for at least 20 generations, are useful in determining the genetic contribution to variability because they are virtually isogenic (i.e., genetically identical). Outbred mice, on the other hand, are a closed population of mice maintained for high heterozygosity for at least 4 generations [21]. Using both inbred and outbred animals, we searched for a low intra-strain variability preserving the possibility of inter-strains differences. The whole gene expression profile in DRG was then assessed to identify a possible correlation between the severity of OIPN and a particular pattern of gene expression modulated by the drug.

This is the first preclinical study aiming at characterizing the susceptibility of different mice strains to oxaliplatin neurotoxicity and correlating it with gene expression study. Significant phenotypic differences in oxaliplatin induced neurotoxicity were demonstrated among strains. Genetic characterization evidenced some gene expression changes that can be considered a first step towards a more comprehensive understanding of a possible role of individual genetic variability.

## Materials and methods

### Animals and husbandry

Three-hundred and thirty-six mice belonging to 1 outbred (CD1) and 5 different inbred (Balb-c, C57BL65, DBA/2J, AJ, FVB) strains aged 10 weeks upon arrival were employed (Envigo, San Pietro al Natisone, Italy).

Animals were housed 5 per cage in a limited access animal facility where room temperature and relative humidity were set at 20 ± 2°C and 55 ± 10% respectively. Artificial lighting provided a 12 h light/12 h dark (7 a.m.–7 p.m.) cycle. Treated and untreated mice were housed separately. The general condition of the animals was assessed daily.

The care and husbandry of animals were in conformity with the institutional guidelines in compliance with national (D. L.vo 26/2014, Gazzetta Ufficiale della Repubblica Italiana, n.61, March 14th 2014) and international laws and policies (European Union directive 2010/63/UE; Guide for the Care and Use of Laboratory Animals, U.S. National Research Council, 1996). The Ethics Committee of the University of Milan Bicocca approved the study plan (n. 004874/14).

All mice were euthanized at the end of the experimental period under deep isoflurane-induced anaesthesia.

### Anaesthesia and euthanasia

For the recordings in the peripheral nerves and in the spinal cord dorsal horn, anaesthesia was induced in a chamber with 3% isoflurane carried in oxygen followed by 1–1.5% isoflurane by nose cone for maintenance during the procedures. The corneal blink response and any withdrawal physical response to external stimuli were adequately suppressed. To avoid isoflurane-induced hypothermia, the body temperature was maintained at ~37°C using a heating pad (Homoeothermic System, Harvard Apparatus, Holliston, MA).

At sacrifice, animals were deeply anesthetized with isoflurane and exanguinated by caval vein puncture.

### Drug

Oxaliplatin (a gift of Debiopharm, Lausanne, Switzerland) solution was prepared as reported by Renn and collaborators [1]. Briefly, oxaliplatin was dissolved in 5% glucose immediately before each administration. It was injected intravenously at the dose of 3.5 mg/kg (10 ml/Kg). Using Du Bois’s formula to calculate human body surface area, this dose is equivalent to 130 mg/m^2^ per administration and a cumulative dose of 1080 mg/m^2^ after 8 cycles.

### Experimental design

Mice of each strain were randomized into a total of 12 groups of 28 animals each. For each strain 28 animals were injected intravenously in the tail vein with oxaliplatin 3.5 mg/Kg (10 ml/Kg) twice a week for 4 weeks (cumulative dose of 28 mg/Kg), and 28 mice were left untreated (naive). Twenty animals (10 naive +10 oxaliplatin-treated) for each strain were employed for neurophysiology, pain behaviour assessment, morphological and morphometric analysis and for platinum concentration. The pool of DRG from other 16 animals (8 naive + 8 oxaliplatin-treated) of each strain were processed for the molecular analysis of gene expression. An independent cohort of 20 animals for each strain (10 naive + 10 oxaliplatin-treated) underwent the electrophysiological analysis in the dorsal horn of the spinal cord (Table 1).

**Table 1:** Experimental design.

STRAINS	NEUROPHYSIOLOGY/BEHAVIOURAL TESTS/IENF/MORPHOLOGY/MORPHOMETRY PLATINUM CONCENTRATION	GENE EXPRESSION	SPINAL ELECTROPHYS.	TOTAL MICE/STRAIN
	20 MICE (NAIVE+OHP)	16 MICE (NAIVE+OHP)	20 MICE (NAIVE+OHP)	56 MICE (NAIVE+OHP)
**Balb-c**	(10+10)	(8+8)	(10+10)	(28+28)
**C57BL6**	(10+10)	(8+8)	(10+10)	(28+28)
**A/J**	(10+10)	(8+8)	(10+10)	(28+28)
**FVB**	(10+10)	(8+8)	(10+10)	(28+28)
**DBA/2J**	(10+10)	(8+8)	(10+10)	(28+28)
**CD1**	(10+10)	(8+8)	(10+10)	(28+28)

The table shows a summary of animals employed for each mice strain and the analysis performed in the study (OHP = oxaliplatin).

Neurophysiological analysis was performed to assess the functionality of peripheral nerves at baseline and at the end of oxaliplatin treatment (week 4). Neuropathic pain was evaluated at baseline, week 2 and week 4 through behavioural tests for cold (acute OIPN) and mechanical (chronic OIPN) thresholds. At week 4, the electrical activity of spinal cord wide dynamic range neurons was determined as a quantitative measure of central nervous system sensitization.

After *in vivo* evaluations, mice were sacrificed for biological sampling: skin biopsy was processed for the evaluation of intraepidermal nerve fibers (IENF) density, caudal and sciatic nerves were harvested for morphological observations and DRG for morphological, morphometric and gene expression analysis of gene arrays and Real Time PCR. Platinum concentration was measured in DRG, sciatic nerve and plasma by atomic absorption spectrometry.

In full respect of the *R*eduction principle of the 3*R*s, the number of animal/group (N = 28) was selected to obtain reliable results and enough biological samples to perform the analysis planned. Moreover, in agreement with the *R*efinement principle, the choice to perform electrophysiological measurements under general anaesthesia reduced to minimum the animal suffering and stress, obtaining less variable data and minimizing the number of necessary animals.

### Characterization of OIPN phenotype in mice

#### Clinical monitoring and body weight

Mice were observed daily for any evident symptoms of sickness. Changes in their appearance (e.g., kyphosis and altered grooming), behaviour (altered nesting) and activity (altered exploring) were monitored. Body weight was recorded twice weekly for general toxicity assessment and drug doses adjustment.

#### Neurophysiology

Forty-eight hours after the end of oxaliplatin treatment, mice were tested for their nerve functionality. Neurophysiology was performed in the caudal and the digital nerves using an electromyography apparatus (Myto2 ABN Neuro, Firenze, Italy) as previously reported [1, 22–24]. Nerve Conduction Velocity (NCV) was measured by placing a couple of needle recording electrodes (cathode and anode) at the base of the tail (for caudal recordings) or at the ankle bone (for digital recordings) and a couple of stimulating electrodes 3.5 cm far from the recording points (for caudal recordings) or close to the fourth toe (for digital recordings). The NCVs were calculated as a ratio between the latency between the stimulus artifact and the onset of the first peak of the elicited action potential and the distance between the recording and the stimulating points. Intensity, duration and frequency of stimulation were set up in order to obtain optimal results. Averaging technique was applied carefully and only when appropriate. For sensory recordings filters were kept between 20 Hz and 3KHz and sweep was kept at 0.5 msec. For both nerves orthodromic stimulation was selected in order to avoid artifacts due to motor activation (particularly in the caudal nerve).

All the neurophysiological measures were obtained under standard conditions in a temperature/humidity controlled rooms. The baseline recordings were performed before starting the drug treatments in order to randomize animals into homogeneous groups. The changes were analysed for significant differences between 2 groups (oxaliplatin-treated vs. naive) of each strain by Student t-test considering p<0.05 as statistically significant.

#### Behavioral tests

At baseline and 24 hours after the 4^th^ and the 8^th^ oxaliplatin treatment, mice were tested for their cold and mechanical thresholds. The cold nociceptive threshold was assessed 24 hours after oxaliplatin, as an indicator of acute response to treatment by using the Cold Plate (model 35100—Hot/Cold Plate, Ugo Basile Biological Instruments, Comerio, Italy) composed by a Plexiglas cylinder and a thermostatic plate, able to reach variable temperatures. Mice were placed on the plate fixed at +4°C, free to move and walk. The number of pain signs/suffering (rear licking and shaking, jumping, alterations in rear and tail movements) were recorded by a blind examiner in a trial of 5 minutes. The trial was anyway terminated if mice prematurely showed a strong intolerance to temperature (evident as anxiety, several changes in the normal behaviour and movements, vocalization).

The mechanical threshold was used to quantify the chronic pain induced by oxaliplatin, using the Dynamic Aesthesiometer (model 37450, Ugo Basile Biological Instruments, Comerio, Italy), which generated a linearly increasing mechanical force. Before testing, animals were placed in a Plexiglas chamber (28 x 40 x 35-cm, wire mesh floor) in the Dynamic Aesthesiometer for a 2 hours acclimatization period. At each time point, a servo-controlled mechanical stimulus (a pointed metallic filament, 0.5 mm diameter) was applied to the plantar surface of the hind paw, which exerted a progressively increasing punctuate pressure, reaching up to 15g within 15 sec. The pressure evoking a clear voluntary hind-paw withdrawal response was recorded automatically and represents the mechanical nociceptive threshold index. The mechanical threshold was expressed as a mean value of 3 measures per hind paw, recorded alternatively every 2 minutes. These results represent the maximal pressure (expressed in grams) tolerated by the animals. The cut-off was set at 30 sec, after which the mechanical stimulus was automatically stopped.

The changes were analysed for significant differences between 2 groups (oxaliplatin-treated vs. naive) of each strain by Student t-test considering p<0.05 statistically significant.

#### Spinal cord electrophysiology

Extracellular electrophysiological recording was performed to measure the activity of wide dynamic range neurons in the spinal dorsal horn as previously described [1, 23]. Briefly, 24 hours after the end of oxaliplatin treatment, mice underwent a laminectomy at the level of the T13-L2 vertebrae, to expose the L4-L5 spinal segment. Dura madre was carefully removed and a tungsten microelectrode (10 μm-tip, Frederick Haer Co., Brunswick, ME, USA) was vertically positioned with an electronical micropositioner at 400–600 μm in depth in the dorsal horn (Model 660 micropositioner, David Kopf Instruments, Tujunga, CA, USA). SciWorks (Datawave Technologies, Loveland, CO, USA) was used to acquire and digitalize the neuronal electrical activity during spontaneous and evoked response by light tactile (sable-hair brush), moderate noxious tactile (press) and thermal (acetone drop) stimulation of the plantar surface of the hindpaw ipsilateral to the electrode position. The signals were amplified and filtered using standard electrophysiological techniques. To optimize the amplitude of an identified neuron, the electrode was moved in the dorsal-ventral plane. Neuronal activity was discriminated, sorted and analysed by principal components analysis offline using SciWorks (v7.0, Datawave Technologies, Berthoud, CO, USA). Stimulus-evoked activity was quantified by calculating the number of spikes/seconds.

The changes were analysed for significant differences between 2 groups (oxaliplatin-treated vs. naive) of each strain by Student t-test considering p<0.05 statistically significant.

#### Neuropathology: Morphological and morphometric assessments

L4-L5 DRG, left sciatic nerves at mid-thigh and caudal nerves at 1 cm far from the base of the tail were dissected out without stretching at sacrifice (48 hours after the last oxaliplatin injection) and fixed by immersion in 2% glutaraldehyde/4% paraformaldehyde or 3% glutaraldehyde in 0.12 M phosphate buffer solutions, respectively. Samples were then post-fixed in OsO_4_, epoxy resin-embedded and used for light microscopy evaluations and morphometric analysis. Semi-thin sections (1 μm) were prepared, stained with toluidine blue and examined with a Nikon Eclipse E200 light microscope (Leica Microsystems GmbH, Wetzlar, Germany) [22, 25]. Serial 1-μm sections, spaced 50 μm, were prepared for the DRG morphometric analysis. Images were captured with a light microscope-incorporated camera (Leica DFC 280 Wetzlar, Germany) at a magnification of 20x. The somatic, nuclear and nucleolar size of at least 200 DRG neurons/animal were manually measured and analysed with a computer-assisted image analyser (Image J software, US National Institutes of Health). The same blinded observer performed all the morphometric measurements [22, 24].

The changes were analysed for significant differences between 2 groups (oxaliplatin-treated vs. naive) of each strain by Student t-test considering p<0.05 statistically significant.

#### IENF density assessment

At sacrifice hind paw skin specimens were collected and processed as previously described [26]. Briefly, glabrous skin punches from the plantar hindpaw were fixed in 2% paraformaldehyde-lysine and periodate sodium for 24 h at 4°C, cryoprotected and serially cut to obtain 20 μm-thick sections. Sections were immunostained with rabbit polyclonal anti-protein gene product 9.5 (PGP 9.5; Bio-Rad Company, AbD Serotec) using a free-floating protocol. The same blinded observer counted the total number of PGP 9.5-positive IENF crossing the dermal–epidermal junction under a light microscope at 40X magnification (Nikon Eclipse E200 light microscope, Leica Microsystems GmbH, Wetzlar, Germany). The linear density of the IENF/mm was calculated [27] accordingly to the length of the epidermis analysed (Microscience Inc., Seattle, WA, USA).

The values were presented as a rate change of oxaliplatin groups compared to their respective naive animals. The changes were analysed for significant differences between 2 groups (oxaliplatin-treated and naive) of each strain by Student t-test considering p<0.05 statistically significant.

#### Platinum concentrations

The total platinum concentration was determined as previously reported by Canta and collaborators [28] on frozen sciatic nerve, DRG, and plasma obtained from 3 animals/group/strain at sacrifice. Frozen samples underwent a digestion process with a HNO-HCL solution and analysed by “Atomic Absorption” (Analyst 600 Perkin Elmer, Monza, Italy). Platinum concentration (μg/g tissue or μg/ml plasma) was calculated accordingly.

### Genotype evaluation: Gene expression analysis

#### Microarray

Gene expression profiling was evaluated for 48 samples from 4 different mouse strains (Balb-c, C57BL6, AJ and CD1). Briefly, total RNA was isolated from sections of frozen DRG from 12 animals (6 animals injected with oxaliplatin 3.5 mg/Kg for 4 weeks and 6 animals untreated) for each strain, using TriReagent^®^ (Sigma, St. Louis, MO, USA). DRGs from each animal were pooled together but each animal was maintained separate for the microarray experiments (i.e. 6 microarrays in the treated group and in the untreated group were analysed for each strain). RNA quantity was evaluated by Bioanalyzer 2100 (Agilent Technologies, Palo Alto, CA, USA). Following the isolation procedure, mRNA was amplified starting from 5 μg of total RNA using MessageAmp aRNA Amplification kit (Ambion Inc., Austin, TX, USA). Amino-allyl modified nucleotides were incorporated during the overnight in vitro transcription step according to the manufacturer’s protocol. Labeling was performed using NHS (N-hydroxysuccinimidyl) ester Cy3 or Cy5 dyes (GE Healthcare Europe GMBH, Upsala, Sweden) able to react with the modified RNA. At least 5 μg of aaRNA for each sample were labeled and then purified with columns; 0.75 μg of labeled aaRNA for each sample were then hybridised. The Dye-Swap replication procedure was applied, in order to increase accuracy. Samples were hybridised on 60K mouse oligo-glass arrays (Agilent Technologies, Santa Clara, California, USA). Arrays were scanned by Agilent scanner. Images obtained were analysed by the Feature Extraction software Agilent (version 9.5) and the text files were then processed using the Bioconductor package Limma (Linear models for microarray analysis). Two-class comparison analysis were performed using the moderated t-statistic available within the Limma package [29]. *P-values* were adjusted for multiple testing by using a false discovery rate (FDR) correction [30]. Analysis then proceeded as described in the main text.

#### Real time quantitative PCR (qRT-PCR)

Total RNA was isolated from sections of frozen DRG from 72 animals using TRI-Reagent^®^ (Sigma Aldrich Inc., Milan, Italy). Specifically, for each strain, 6 control and 6 treated animals were used. mRNA from different animals was not pooled and therefore each mRNA was analysed separately. Im-Prom-II^™^ Reverse Transcriptase (Im-Prom-II^™^ Reverse Transcription System, Promega, WI, USA) was used to generate cDNA using 1 μg of RNA and oligo dT primers, according to the instructions of the manufacturer. Reverse transcription was performed using a thermal-cycler (Eppendorf, Hamburg, Germany) and the following heating protocol: 10 minutes at 25°C, 45 minutes at 42°C and 5 minutes at 99°C. cDNA was then stored at -20°C until further used. qRT-PCRs were performed on a 96-well plate, in triplicate, and florescence intensity assessed using the CFX96^™^ Real-Time PCR Detection Systems (Bio-Rad Inc., Milan, Italy). The following conditions were adopted: 12.5 μL Maxima^™^ SYBR Green/ROX qPCR Master Mix (Thermo Fisher Scientific Inc., Milan, Italy), 0,1 μM of forward and reverse primers, and 5 μL of 1:5 diluted cDNA, in a total volume of 25 μL/reaction. The list of primers used is in S1 Table. Transcripts were normalized to the expression of Ribosomal Protein S18 mRNAs, assessed using the 5’-TGCGAGGTACTCAACACCAACA-3’ FW and 5’-CTGCTTTCCTCAACACCACA-3’ RV primers (Sigma-Aldrich Inc., Milan, Italy), 60°C annealing temperature. For each gene, the threshold cycle *(C*_*t*_*)* was calculated using CFX Manager Software (Bio-Rad Inc., Milan, Italy) by determining the cycle number at which the change in the fluorescence of the reporter dye crossed the threshold. The *C*_*t*_ of treated cells was compared to the *C*_*t*_ generated by the control cells and *ΔC*_*t*_ was calculated as the difference between *C*_*t*_ values, determined using the equation 2-^ΔCt^.

## Results

### Acute and chronic oxaliplatin neurotoxicity

Oxaliplatin chronic treatment was generally well tolerated by the animals of all strains. Mice continued to groom, eat, drink and explore their surroundings. No animal died during the treatment, body weight loss was below 10% (data not presented) and only approximately 20% of animals showed signs of mild kyphosis and piloerection.

The acute symptoms of neurotoxicity were assessed by Cold Plate test.

Then, we employed neurophysiological and behavioural analysis, neuropathological evaluations of the DRG, sciatic and caudal nerve and morphometric measures of DRG sensory neurons and of IENF to study the extent of functional and structural damages induced by chronic oxaliplatin treatment.

#### Cold Plate test

**Fig 1:**
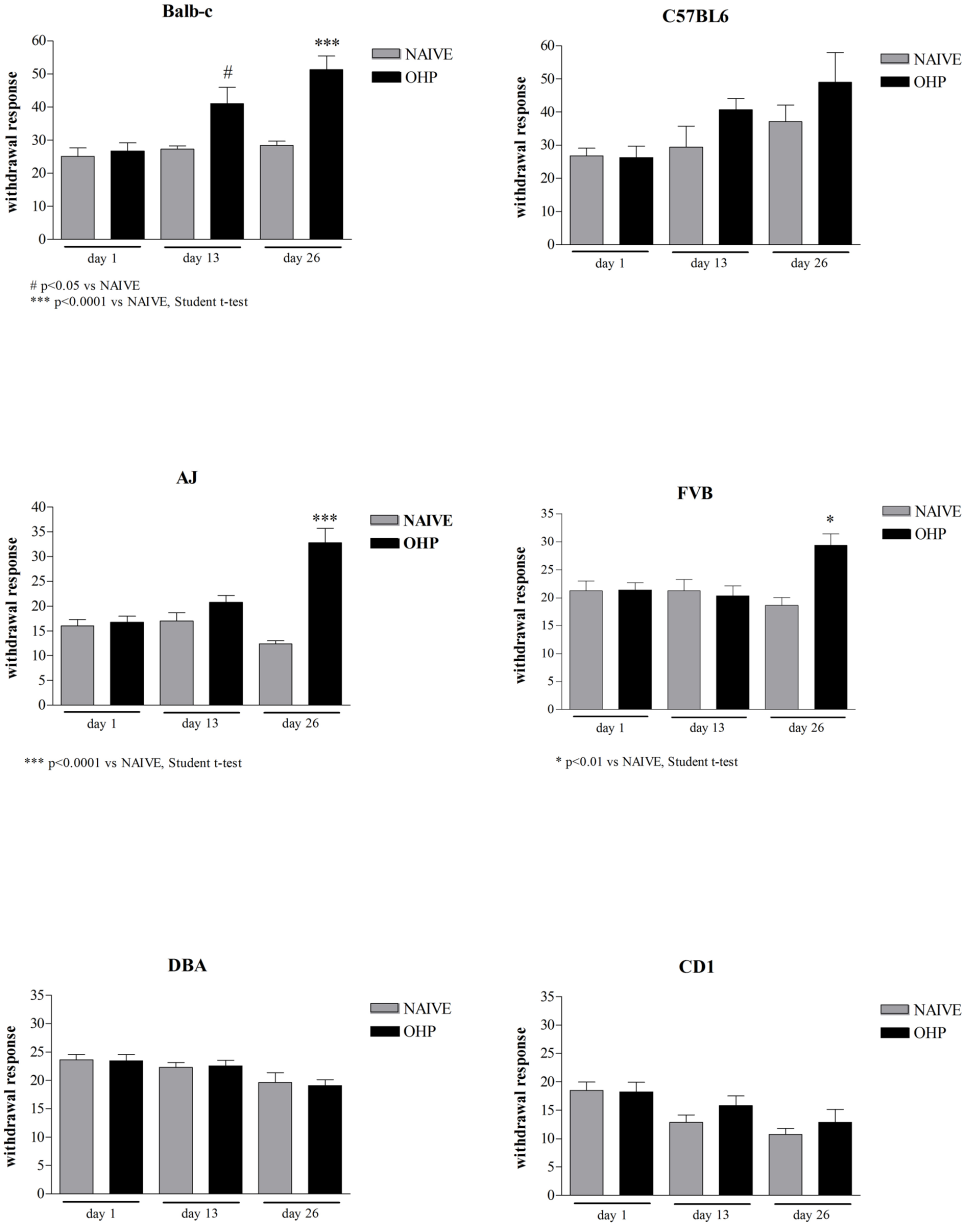
Cold Plate test. The graphs report the number of events related to pain (withdrawal response) during thermal (cold) stimulation of oxaliplatin-treated animals versus naive animals at baseline, day 13 and day 26.

Beyond the chronic functional and structural abnormalities in the peripheral nervous system, oxaliplatin treatment firstly caused an acute hypersensitivity to cold exposure within 48 hours after infusion. To determine whether different strains differently exhibit signs of cold hyperalgesia, mice were tested for their responses to cold stimulations at baseline (day 1), 24 hours after the 4^th^ (day 13) and 24 hours after the 8^th^ oxaliplatin injection (day 26). Cold hyperalgesia was defined as an increase in the sensitivity of animals to a nociceptive cold temperature (+4°C) recorded as a number of events related to pain (jumping, licking etc.) in a given time. Twenty-four hours after the last oxaliplatin injection Balb-c, AJ and FVB, but not C57BL6, DBA/2J and CD1 mice had a statistically significant increase of the number of events related to pain 24 hours after oxaliplatin injection (80.6%, p<0.001 vs NAIVE for Balb-c; 31.9%, p = NS for C57BL6; 51.5%, p<0.01 vs NAIVE for FVB; 165%, p<0.0001 vs NAIVE for AJ; 3.1%, p = NS for DBA/2J and 19.6%, p = NS for CD1). (Fig 1).

#### Neurophysiology

To assess the functional status of peripheral nerves, NCVs were measured in the caudal and digital nerves 3 days after the last oxaliplatin dose. Table 2 shows the mean NCV values (± SD) of oxaliplatin-treated groups and their naive counterparts, the rate change of treated animals compared to untreated mice and the statistical analysis. Only Balb-c and FVB mice showed a statistically significant reduction of both caudal and digital NCV (Balb-c: -13% and -14.7% for caudal and digital respectively, p<0.0001 vs NAIVE; FVB: -12.6% and -13% for caudal and digital respectively, p<0.001 vs NAIVE). Oxaliplatin-treated DBA/2J showed a significant 8% decrease in the caudal NCV (p<0.001 vs NAIVE) while only digital NCV was decreased in CD1 mice (-22%, p<0.0001 vs NAIVE). No significant functional changes were recorded in the peripheral nerves of C57BL6 and AJ mice.

**Table 2:** Nerve Conduction Velocity (NCV).

	**CAUDAL NCV (m/sec)**		
	**NAIVE**	**OHP**	**CHANGE vs NAIVE (%)**
**Balb-c**	27.1±0.37	23.6±0.40 (***)	-13.0
**C57BL6**	26.7±0.42	26.3±0.48	-1.6
**AJ**	21.8±0.44	22.1±0.56	1.4
**FVB**	29.1±0.59	25.5±0.63 (**)	-12.6
**DBA/2J**	28.3±0.27	26.0±0.45 (**)	-8.0
**CD1**	25.4±0.33	24.0±0.58	-5.4
	**DIGITAL NCV (m/sec)**		
	**NAIVE**	**OHP**	**CHANGE vs NAIVE (%)**
**Balb-c**	28.5±0.51	24.3±0.53 (***)	-14.7
**C57BL6**	27.2±0.56	27.2±0.57	-0.3
**AJ**	25.6±0.96	23.2±0.85	-9.3
**FVB**	28.8±0.65	25.0±0.49 (**)	-13.0
**DBA/2J**	27.0±0.66	28.0±0.82	3.5
**CD1**	25.1±0.80	19.5±0.35 (***)	-22.0

***p<0.0001 vs NAIVE;

**p<0.001 vs NAIVE; Student t test

#### Morphological analysis of DRG, sciatic nerve and skin biopsies

**Fig 2:**
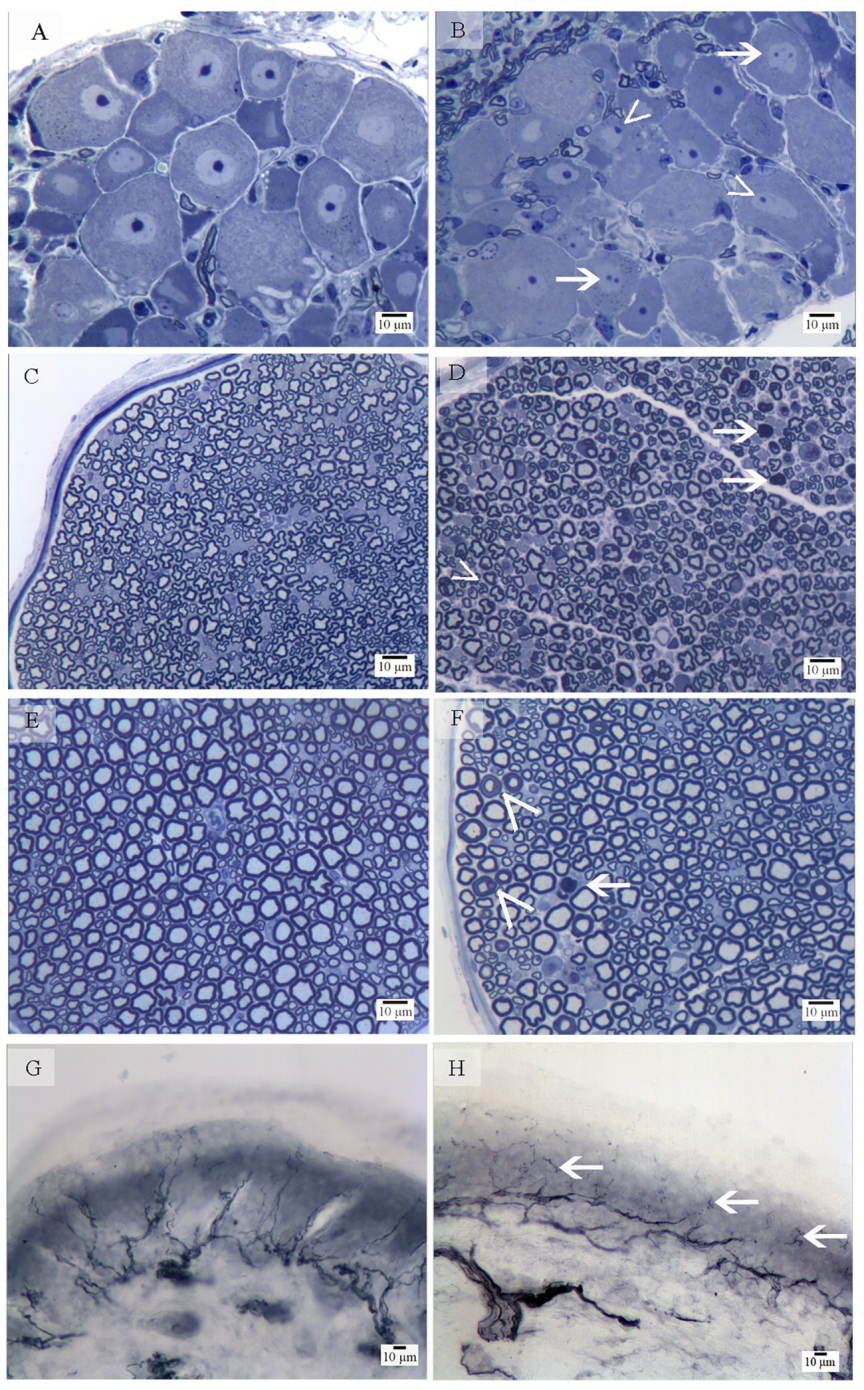
Morphological analysis at light microscope. Naive DRG (A), caudal nerve (C), sciatic nerve (E) and skin (G) were compared to oxaliplatin treated ones (B, D, F, H) of Balb-c mice (explanatory of the neurotoxic damage). B: multiple (arrows) and eccentric (arrowhead) nucleoli can be observed; D and F: degenerating fibers (arrows) and myelin derangement (arrowheads) are indicated; H: arrows point at the site of unmyelinated fiber density reduction in the epidermis.

Next, we examined structural changes in DRG, sciatic and caudal nerves and in unmyelinated fibers of the skin. In Fig 2 we reported representative pictures of DRG (A-B), peripheral nerves (caudal nerves, C-D and sciatic nerves, E-F) and skin biopsies (G-H) of Balb-c mice. Oxaliplatin caused typical morphological alterations in DRG sensory neurons at the light microscope, as previously reported [1, 31] [32]: the increased incidence of multiple and eccentric nucleoli (Fig 2B) was evident in treated animals compared to naive (Fig 2A). A moderate number of degenerating nerve fibers (Fig 2D) and a slight fiber loss were observed in the caudal nerve of Balb-c mice treated with oxaliplatin. Degenerating fibers (Fig 2F), indicative of a slight axonopathy, were evident in sciatic nerves of Balb-c animals treated with oxaliplatin compared to their naive (Fig 2C and 2E). Very mild myelin derangement (Fig 2F) represented by uncompacted sheats was only occasionally seen. The morphological aspect of the unmyelinated fibers in the skin biopsies (IENF) was normal, despite a severe reduction in their density (Fig 2H), in animals treated with oxaliplatin compared to the naive (Fig 2G). Similar, but milder, changes were evident in the other strains.

#### Morphometric analysis of DRG neurons

To further investigate the structural changes in the DRG, we performed a morphometric analysis of DRG neurons. The mean values (± SD) of somatic, nuclear and nucleolar areas of oxaliplatin-treated groups and their naïve counterparts, the rate change of treated animals compared to the untreated mice and the statistical analysis can be found in Table 3. The results of this quantitative analysis support the neurophysiological data: only oxaliplatin-treated Balb-c and FVB mice showed a severe and statistically significant reduction in size of all the neuronal structures compared to their naive counterparts (Balb-c: -12.6%, p<0.001 vs NAIVE for somatic area, -11.9% and -31.8%, p<0.0001 vs NAIVE for nuclear and nucleolar areas; FVB: -9.9% and -12.9% p<0.001 vs NAIVE for somatic and nucleolar areas, -10.1%, p<0.0001 vs NAIVE for nuclear area). Oxaliplatin-treated AJ, DBA/2J and CD1 mice showed significant reduction in size of 1 or 2 cellular structures analysed while drug-treated C57BL6 mice DRG were not different from their naïve controls (Table 3).

**Table 3:** DRG and IENF morphometric analysis.

	**DRG SOMATIC AREA (μm**^**2**^**)**		
	**NAIVE**	**OHP**	**CHANGE vs NAIVE (%)**
**Balb-c**	609.9 ± 15.81	533.1 ± 13.22 (**)	-12.6
**C57BL6**	546.1 ± 14.80	542.3 ± 13.34	-0.5
**AJ**	584.6 ± 15.89	554.0 ± 15.33	-5.1
**FVB**	546.1 ± 13.85	492.8 ± 11.83 (**)	-9.9
**DBA/2J**	631.4 ± 18.09	576.6 ± 15.61 (#)	-8.7
**CD1**	594.2 ± 14.25	538.3 ± 14.13 (**)	-9.4
	**DRG NUCLEAR AREA (μm**^**2**^**)**		
	**NAIVE**	**OHP**	**CHANGE vs NAIVE (%)**
**Balb-c**	101.6 ± 1.91	89.4 ± 1.74 (***)	-11.9
**C57BL6**	91.6 ± 1.78	89.9 ± 1.69	-1.9
**AJ**	97.1 ± 1.87	96.6 ± 1.83	-0.6
**FVB**	95.1 ± 1.94	85.6 ± 1.41(***)	-10.1
**DBA/2J**	97.9 ± 1.90	94.8 ± 1.70	-3.5
**CD1**	97.2 ± 1.82	90.8 ± 1.63 (**)	-6.6
	**DRG NUCLEOLAR AREA (μm**^**2**^**)**		
	**NAIVE**	**OHP**	**CHANGE vs NAIVE (%)**
**Balb-c**	7.9 ± 0.21	6.77 ± 0.18 (***)	-31.8
**C57BL6**	6.3 ± 0.16	6.25 ± 0.15	-2.2
**AJ**	7.2 ± 0.19	5.99 ± 0.16 (***)	-17.8
**FVB**	8.7 ± 0.27	7.63 ± 0.18 (**)	-12.9
**DBA/2J**	7.8 ± 0.20	6.91 ± 0.16 (**)	-12.2
**CD1**	6.7 ± 0.17	6.77 ± 0.16	0.1
	**IENF (fibers/mm)**		
	**NAIVE**	**OHP**	**CHANGE vs NAIVE (%)**
**Balb-c**	45.2±2.42	29.2±2.08 (**)	-35.5
**C57BL6**	34.7±20.9	35.2±2.70	1.38
**AJ**	31.2±0.82	26.7±1.44 (#)	-14.5
**FVB**	31.0±1.05	31.4±1.33	1.4
**DBA/2J**	35.6±1.79	29.7±1.66 (#)	-24.0
**CD1**	27.1±1.10	23.0±1.58 (*)	-15.1

The table reports the mean ± SD of the somatic, nuclear and nucleolar areas (μm^2^) of at least 200 sensory neurons of DRG and the linear density of IENF expressed as mean ± SD of the unmyelinated fibers/mm in naive and oxaliplatin-treated animals of each strain. The change (%) in oxaliplatin-treated versus respective naive animals is also reported.

DRG: #p<0.05 vs NAIVE; **p<0.001 vs NAIVE; ***p<0.0001 vs NAIVE; Student t test

IENF: **p<0.001; *p<0.01; #p<0.05 vs NAIVE; Student t test

#### Morphometric analysis of unmyelinated fibers in skin biopsies

IENF density was assessed to determine the neurotoxic injury on small fibers after oxaliplatin chronic treatment [33–35]. The mean values (± SD) of IENF in oxaliplatin-treated groups and their naive counterparts, the rate change of treated animals compared to the untreated mice and the statistical analysis can be found in Table 3. The morphometric assessments performed on skin biopsies of animals belonging to all mice strains demonstrated a statistically significant reduction of IENF in Balb-c, AJ, CD1 and DBA/2J treated with oxaliplatin compared to their controls (-35.5%, p<0.001 vs NAIVE for Balb-c; -15.1%, p<0.01 vs NAIVE for CD1; -14.5%, p<0.05 vs NAIVE for AJ and –24%, p<0.05 vs NAIVE for DBA/2J) (Table 3).

### Neuropathic pain assessment

#### Dynamic test for mechanical allodynia

**Fig 3:**
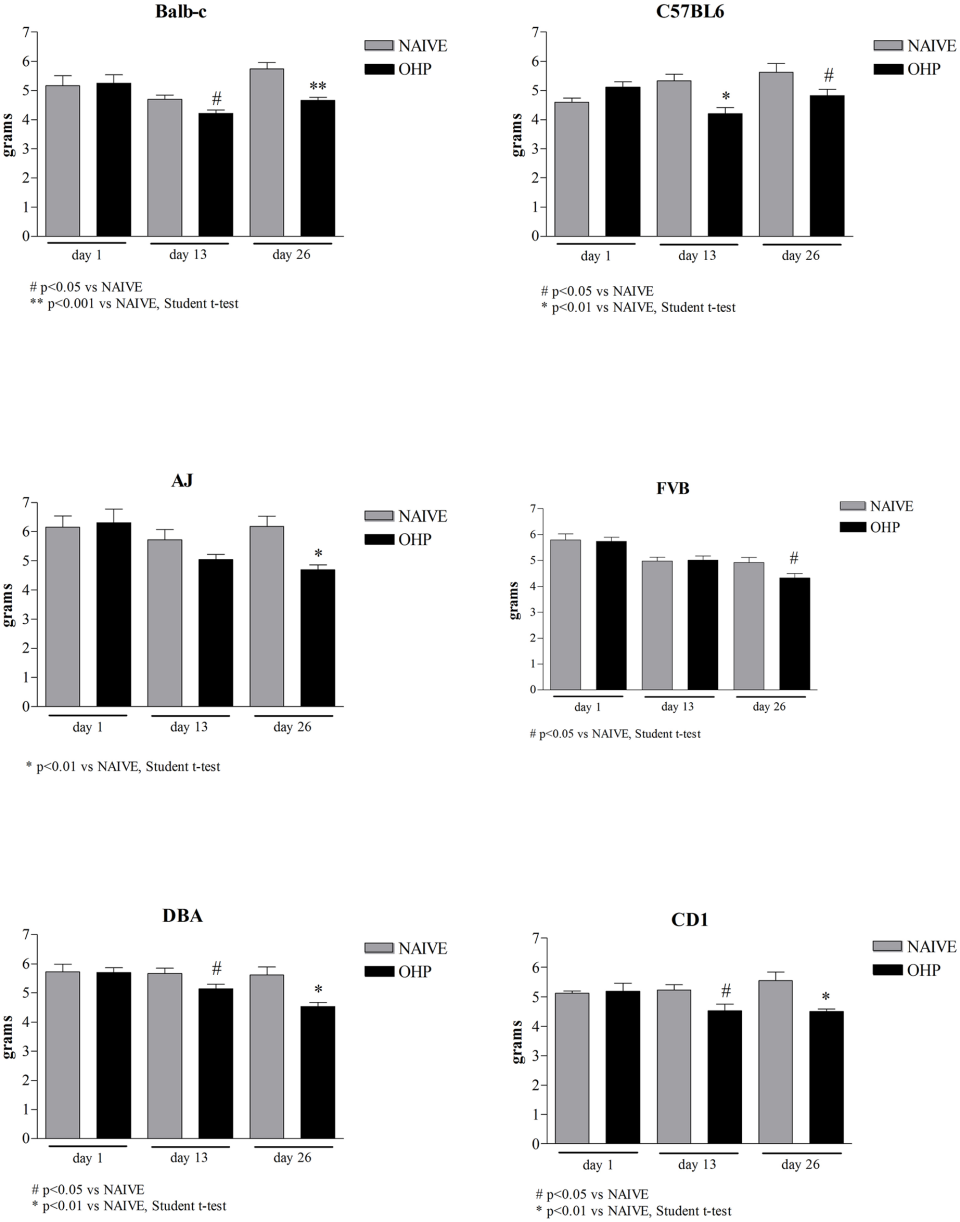
Dynamic test. The graphs report the pressure (grams) evoking a withdrawal response during mechanical stimuli of oxaliplatin-treated animals versus naive animals at baseline, day 13 and day 26.

As widely observed in cancer patients and reproduced in a few animal models [1, 36], oxaliplatin treatment causes peripheral neurotoxicity, and neuropathic pain after chronic treatments. To determine whether our strains of mice differently exhibit changes in the sensory threshold, animals were tested for mechanical sensitivity. Mice were tested at baseline (day 1), 24 hours after the 4^th^ (day 13) and the 8^th^ oxaliplatin injection (day 26). Mice in all strains showed a decrease in the withdrawal response (grams) during a non-noxious punctate mechanical pressure on the surface of the hind paw both at day 13 and 26 (mechanical allodynia), with a different statistical significance. Twenty-four hours after the last oxaliplatin injection Balb-c, C57BL6, AJ, FVB, DBA/2J and CD1 mice reached respectively a 19%, 14.3%, 24%, 9.2%, 19.5% and 18.9%-significant decrease in their mechanical threshold compared to their respective naive animals (p<0.001 vs NAIVE for Balb-c; p<0.01 vs NAIVE for AJ, DBA/2J and CD1; p<0.05 vs NAIVE for C57BL6 and FVB) (Fig 3).

#### Electrophysiological analysis of wide dynamic range (WDR) neurons in the spinal dorsal horn

It was previously demonstrated that oxaliplatin is able to induce physiological changes in the primary afferents as well as in the spinal cord dorsal horn WDR neurons in Balb-c mice [1]. WDR neurons produces an electrical response to innocuous (i.e brush), noxious (i.e press) and thermal (i.e. acetone) stimulation applied to the plantar surface of the hind paw ipsilateral to the recording site in the spinal cord. It is hypothesized that, after the exposure to oxaliplatin, increased excitatory activity of central neurons is secondary to peripheral sensitization of primary afferents.

Twenty-four hours after the last oxaliplatin dose, cohorts of dedicated mice underwent the surgical procedure to expose the spinal cord lumbar segment. The activity was recorded from a mean of 15 WDR neurons/animal from 10 animals/group. The mean values (± SD) of the number of spikes/second recorded during 10 seconds stimulation with brush, 2 seconds stimulation with press and 2 seconds stimulation with cold acetone in oxaliplatin-treated animals and in their naive counterparts, the rate change of treated animals compared to the untreated mice and the statistical analysis can be found in S2 Table.

In agreement with the results of the behavioural tests, oxaliplatin-treated Balb-c and AJ mice (that were particularly affected by mechanical allodynia and acute cold thermal hyperalgesia) showed a number of spikes significantly higher during stimulation with the innocuous brush (Balb c:+40.2% p<0.05 vs NAIVE; AJ +238%, p<0.0001 vs NAIVE), the moderately noxious press (Balb-c +102%, p<0.05 vs NAIVE; AJ: +119%, p<0.01 vs NAIVE) and the cold acetone (Balb-c: +93.4%, p<0.05 vs NAIVE; AJ: +297%, p<0.001 vs NAIVE). FVB animals that developed acute cold hyperalgesia 24 hours after the last oxaliplatin treatment and mechanical allodynia did not show any sign of increased spinal dorsal horn neuronal activity during mechanical stimulations, but had a significant increase in the number of spikes/second during the cold thermal stimulation with acetone (+132%, p<0.05 vs NAIVE). C57BL6 mice, which showed only mechanical allodynia, had increased spinal dorsal horn neuronal activity with both the innocuous brush and cold acetone (+44%, p<0.05 vs NAÏVE and (+111%, p<0.01 vs NAÏVE, respectively).

All the results reported so far are summarized in Table 4.

**Table 4:** Summary of the results of neurotoxicity studies.

	STRAINS					
	Balb-c	C57BL6	AJ	FVB	DBA/2J	CD1
**PERIPHERAL NEUROPATHY AND GANGLIONOPATHY**						
**ACUTE NEUROTOXICITY** **(COLD HYPERALGESIA)**	**		***	*		
**NEUROPHYSIOLOGY** **(CAUDAL NCV)**	***			**	**	
**NEUROPHYSIOLOGY** **(DIGITAL NCV)**	***			**		***
**DRG MORPHOMETRY** **(SOMA)**	**			**	#	**
**DRG MORPHOMETRY** **(NUCLEUS)**	***			***		**
**DRG MORPHOMETRY** **(NUCLEOLUS)**	***		***	**	**	
**IENF DENSITY**	**		#		#	*
**NEUROPATHIC PAIN**						
**MECHANICAL ALLODYNIA**	**	#	*	#	*	*
**ELECTROPHYSIOLOGY** **(BRUSH)**	#	#	***			ND
**ELECTROPHYSIOLOGY** **(PRESS)**	#		*			ND
**ELECTROPHYSIOLOGY** **(ACETONE)**	#	*	#	#		ND

The table summarizes the results of the statistical analysis performed on each parameter evaluated to assess the severity of peripheral neuropathy and neuropathic pain induced by oxaliplatin in mice strains.

***p<0.0001;

**p<0.001;

*p<0.01;

^#^p<0.05 oxaliplatin-treated vs. NAIVE;

Student t test

### Platinum concentration

Platinum concentration was measured by atomic absorption 72 hours after the last oxaliplatin injection in DRG, sciatic nerves and plasma of oxaliplatin-treated animals of each strain. Platinum concentration in plasma was similar, but irrelevant in all strains confirming that the drug clearance was almost completed within 48 hours. The concentration of platinum stored in DRG and sciatic nerves was very high in all the strains considered. The mean amount of platinum measured in the DRG of AJ mice was significantly higher compared to Balb-c (p<0.05 vs AJ), C57BL6, FVB and DBA/2J (p<0.001 vs AJ). No statistically significant differences between strains were detected in the sciatic nerves.

The data on platinum concentration can be found in S3 Table.

### Gene expression evaluation

We then performed microarray analysis of DRG from AJ, CD1, Balb-c and C57BL6 mice to evaluate gene expression changes. Analysis was performed comparing gene expression changes among samples from treated and untreated animals. We included in the analysis only genes with an *average expression* greater than 6 (log2 Intensity values) and showing a ǀlog2FCǀ > 0.5, as lower expressions are, in our experience, unreliable. Eight genes were significantly up-regulated and 14 were significantly down-regulated in Balb-c-treated animals, 3 genes were up-regulated and 11 were down-regulated in AJ-treated animals, 1 gene was up-regulated and 4 genes were down-regulated in CD1-treated mice. No genes were significantly changed in C57BL6. This last observation, surprisingly, contrasts with the significant effect of OHP on mechanical allodynia and WDR neurons in the spinal cord. Most genes were differentially expressed in a single mouse strain although some common features were observed (Table 5, S1 Fig). Most changes were of moderate intensity although CD74 and H2-Eb1 in Balb-c and H2-Ab1 in CD1 showed log2 fold changes greater than 2. All other changes showed a log2 fold-change of about 1.

**Table 5:** List of genes that displayed significant changes between treated and untreated mice.

	**Balb-c**					
	**ProbeName**	**GeneSymbol**	**p-values**	**adj.p-values**	**log_FC**	**AveExpr**
**Up-regulated**	A_55_P1987499	Pttg1	0.000000	0.001638	1.136716	11.216251
	A_55_P1959500	Ces2e	0.000001	0.003382	1.102319	6.898396
	A_55_P1965154	Spc25	0.000037	0.043139	0.722801	8.389159
	A_51_P457528	Ccnb2	0.000047	0.047962	0.702836	7.729787
	A_55_P1960735	Gdf15	0.000000	0.000146	0.606808	6.644995
	A_51_P246339	Rfc5	0.000044	0.047510	0.520725	9.200125
	A_51_P455897	Fam64a	0.000024	0.036096	0.505713	6.398450
**Down-regulated**	A_51_P223498	Slc39a10	0.000023	0.036096	-0.556914	8.941834
	A_66_P109002	Kcng2	0.000011	0.025093	-0.801808	8.403939
	A_51_P222741	H2-Ea-ps	0.000000	0.000000	-0.869288	6.206409
	A_52_P99810	Cx3cr1	0.000002	0.011096	-0.881694	7.221797
	A_55_P1964363	Kctd14	0.000030	0.040871	-0.914326	8.966172
	A_55_P2104412	Gm10081	0.000038	0.043139	-0.997577	6.375892
	A_52_P574274	Spatc1	0.000000	0.002257	-1.093003	6.606756
	A_55_P2165790	Siglech	0.000000	0.000247	-1.106950	6.821341
	A_52_P1135722	Gm684	0.000014	0.027716	-1.277763	6.766403
	A_51_P278868	H2-DMb1	0.000000	0.000212	-1.279237	9.457730
	A_55_P1962747	H2-Ab1	0.000001	0.003914	-1.578391	11.720059
	A_55_P2146560	H2-Ab1	0.000000	0.001134	-1.709102	11.478677
	A_55_P2156731	H2-Eb1	0.000000	0.000064	-2.241588	11.624532
	A_51_P284608	Cd74	0.000000	0.000135	-2.477466	10.681315
	**AJ**					
	**ProbeName**	**GeneSymbol**	**p-values**	**adj.p-values**	**log_FC**	**AveExpr**
**Up-regulated**	A_55_P1976694	Sept11	0.000013	0.040107	0.650315	9.273354
	A_55_P2141013	Siglech	0.000000	0.002661	0.636849	15.720079
	A_55_P1982737	Dnajc5	0.000005	0.022082	0.571697	15.771884
**Down-regulated**	A_51_P370678	Gfi1b	0.000015	0.040107	-0.521881	6.599796
	A_55_P2153382	Ermap	0.000002	0.014967	-0.530305	6.268419
	A_55_P2082929	H2-Ob	0.000011	0.040107	-0.633375	6.475577
	A_51_P231320	Mmp8	0.000015	0.040107	-0.819533	6.533332
	A_55_P2091551	Arhgap9	0.000010	0.040107	-0.824860	8.352636
	A_51_P194609	Prss34	0.000001	0.011873	-0.871312	6.516110
	A_51_P391716	Ermap	0.000001	0.011873	-0.911140	6.493820
	A_55_P1997126	Ctse	0.000002	0.014967	-1.045790	6.492716
	A_51_P299062	Kel	0.000001	0.011873	-1.205625	6.585137
	A_55_P2165790	Siglech	0.000003	0.021889	-1.408224	7.074684
	A_51_P241769	Rhd	0.000000	0.002661	-1.420892	6.805098
	**CD1**					
	**ProbeName**	**GeneSymbol**	**p-values**	**adj.p-values**	**log_FC**	**AveExpr**
**Up-regulated**	A_51_P168792	Xylb	0.000007	0.041672	0.544057	8.731946
**Down-regulated**	A_51_P496804	Adat2	0.000004	0.034816	-0.845346	9.166717
	A_51_P284608	Cd74	0.000001	0.021062	-2.443737	10.825574
	A_55_P1962747	H2-Ab1	0.000000	0.000022	-2.641439	11.335051
	A_55_P2146560	H2-Ab1	0.000000	0.000029	-3.123013	10.931527

The observation that different genes were modified by oxaliplatin in the different mouse strains, together with the small changes observed, led us to hypothesize that the protocol employed possibly had low statistical power to determine small-to-medium-changes and that variability within samples of the same strain also was a confounding factor. This hypothesis, together with the complex behavioural phenotypes described above, prevented us from correlating single genes to single observations.

To circumvent in part these limitations, we next decided to perform a supervised analysis of the results, independently of statistical significance, as a pilot investigation. Again, we included in the analysis only genes with an *average expression* greater than 6 (log2 Intensity values). Secondly, we extracted only those genes that the microarray projected as doubling or halving their levels upon treatment (a logFC >1 or <-1) in at least one strain. This led to a total of 122 up-regulated genes and 103 down-regulated genes (S4 Table).

As can be observed from the Venn diagram (S1 Fig), genes were again modulated differently in the different strains. However, we felt that, while of possible interest, the lack of statistical significance for most of these genes and the post-hoc nature of this analysis did not allow us to turn our attention to single genes that displayed a different behaviour in the different strains.

On the contrary, we felt that the 8 genes with a fold-change of logFC >1 (Tnnc2, Myl1, Pgam, Myh8, Tnni2, Tnnt3 v1) or <-1 (H2-Eb1, CD74) in all 4 strains deserved further attention. Furthermore, we implemented the list with a further 12 genes in which this fold-change was verified in at least 2 strains and was > 0.5 (Myh4, Tcap, Cox6a2, Tnnt3 v7, Acta1, Mb v2, Myl2, Ckm, Myl1 v3, Mybpc2) or<-0.5 (H2-Ab1, Siglech) in the other 2 strains. Due to the heuristic nature of the pure logFC criterion, we decided to support the selection of the common differentially regulated genes by means a statistically rigorous approach known as the rank product method. This algorithm, firstly reported by Breitling et al. ^17^ and Heskes et al. ^18^ is a non-parametric statistical test based on ranks of FC (or logFC) expression values. The procedure (S1 File), validated the former logFC-based gene list. We implemented, as a control, this list with the 8 genes with |log2FC|>1 that had shown significant statistical changes in one mouse strain but that were not included in the above list as a trend of change was not observed in the other mouse strains.

**Fig 4:**
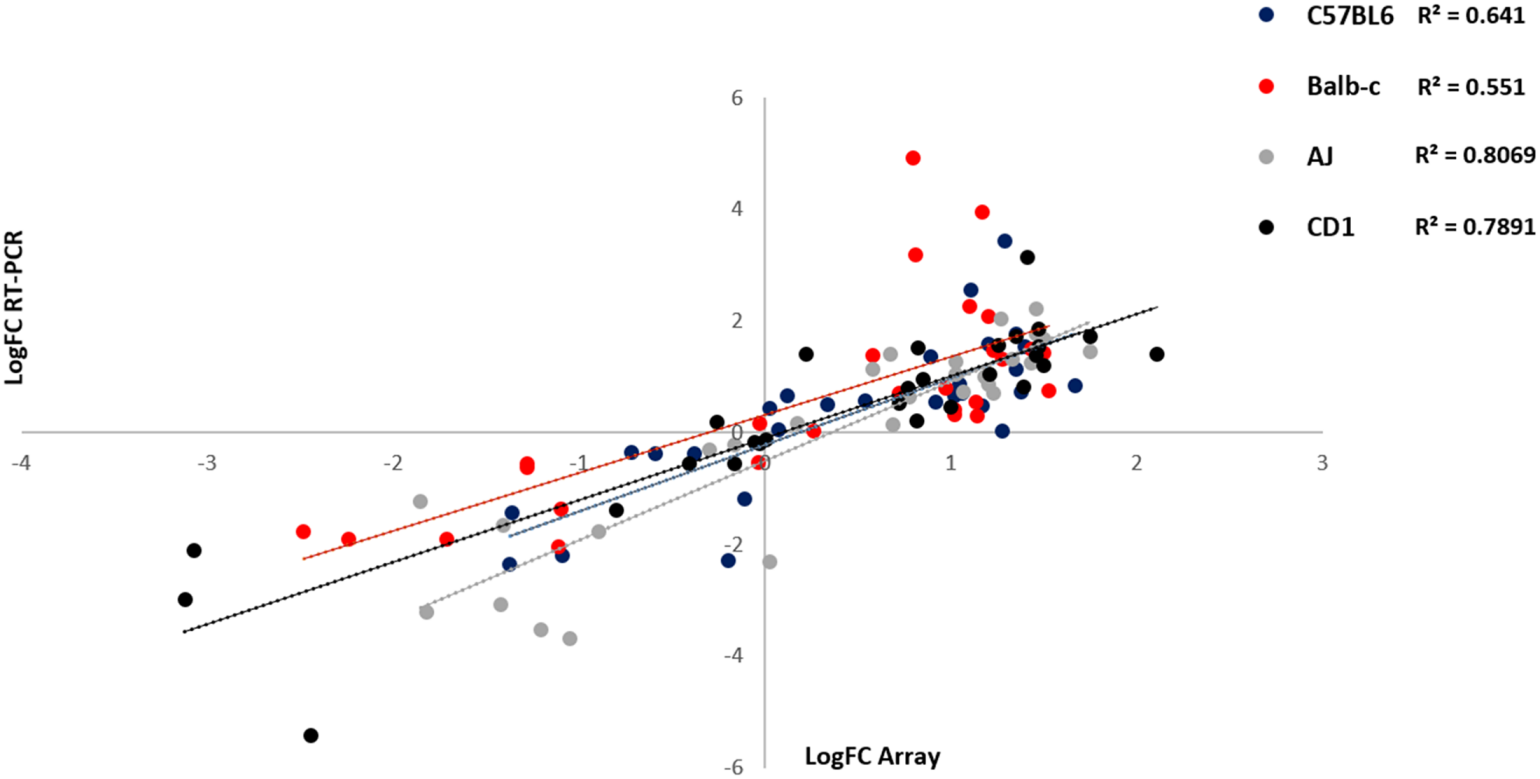
Correlation of the log-change of the 30 selected genes in the 4 strains as predicted by microarray analysis and RT-PCR.

First, we used these genes to confirm whether the data from the microarray experiments were reliable, validating them using a different set of treated and untreated tissues and analysing the relative abundance of the genes by RT-PCR. A gross analysis of the results revealed a good correlation between the data obtained by microarray and the data obtained by RT-PCR (Fig 4). This showed, therefore, that the changes observed in the microarrays could be grossly replicated by other methods. This was confirmed by the fact that when analysing the single genes that had met the criteria above, 15 out of the 20 analysed transcripts were confirmed to increase or decrease of at least 1.5-fold compared to control untreated tissues in all 4 strains (Table 6). On the other hand, only 1 of the 8 genes (Ctse) that had shown statistical significance in a single strain, but that had not met the above criteria, met the criteria in the four strains in RT-PCR experiments.

**Table 6:** Genes evaluated in RT-PCR experiments.

	C57BL6	Balb-c	AJ	CD1	DBA/2J	FVB
**Tnnc2**	**31.00±0.47**	**2.13±0.62**	**4.25±0.43**	**5.6±0.47**	**4.31±0.27**	**3.75±0.06**
**Myl1**	3.13±0.51	4.13±0.63	5.85±0.76	4.69±0.98	1.38±0.29	4.03±0.36
**Pgam**	2.02±0.65	1.34±0.41	2.39±0.43	2.83±0.34	1.66±0.19	16.36±0.66
**Myh8**	12.87±0.99	4.39±0.39	3.74±0.14	4.00±0.63	0.34±0.08	1.38±0.33
**Tnni2**	1.63±0.28	7.94±0.25	2.09±0.34	23.02±0.55	0.59±0.11	0.36±0.099
**Tnnt3 v1**	5.85±0.86	1.39±0.93	2.05±0.51	2.28±0.51	1.17±0.21	0.91±0.068
**H2-Eb1** (a)	**0.095±0.0063**	**0.15±0.02**	**0.29±0.03**	**0.12±0.006**	**0.91±0.036**	**0.18±0.012**
**CD74** (b)	**0.11±0.04**	**0.17±0.06**	**0.04±0.006**	**0.0044±0.001**	**0.03±0.001**	**0.10±0.06**
**Myh4**	**4.94±0.97**	**2.2±0.50**	**9.19±0.29**	**6.34±0.38**	**3.76±0.56**	**3.39±0.12**
**Tcap**	1.74±0.45	4.44±0.52	7.59±0.45	3.32±0.60	0.20±0.001	3.04±0.55
**Cox6a2**	1.94±0.65	1.52±0.01	2.86±0.79	4.81±0.43	2.34±0.67	0.99±0.034
**Tnnt3 v7**	2.06±0.86	136.87±0.37	3.09±0.46	5.65±0.47	0.98±0.05	2.62±0.38
**Acta1**	2.34±0.58	4.12±0.97	3.53±0.42	2.24±0.42	0.07±0.03	2.79±0.44
**Mb v2**	3.92±0.45	3.76±0.98	1.90±0.55	4.08±0.59	0.18±0.017	3.26±0.14
**Myl2**	1.78±0.61	51.98±0.71	1.16±0.004	1.59±0.40	0.08±0.004	2.31±0.17
**Ckm**	4.66±0.058	24.45±0.39	5.32±0.56	2.60±0.56	1.33±0.23	1.04±0.098
**Myl1 v3**	1.04±0.12	2.04±0.36	3.45±0.36	1.68±0.61	1.00±0.012	0.77±0.045
**Mybpc2**	2.35±0.25	4.00±1.05	2.74±0.29	1.24±0.46	0.70±0.2	1.52±0.078
**H2-Ab1** (b)	0.24±0.003	0.15±0.0002	0.17±0.035	0.05±0.0001	0.86±0.47	0.22±0.071
**Siglech** (c)	**0.70±0.12**	**0.13±0.02**	**0.19±0.032**	**0.25±0.07**	**0.18±0.08**	**0.17±0.067**
**Ctse** (d)	0.67±0.36	0.59±0.08	0.025±0.08	0.58±0.057	5.45±0.95	0.73±0.068
**Pttg1** (a)	1.06±0.10	1.72±0.47	0.73±0.061	9.04±1.93	5.14±0.94	0.63±0.039
**Gm684** (a)	1.94±0.16	0.54±0.077	0.81±0.067	0.57±0.016	1.32±0.26	0.75±0.021
**Rhd** (d)	1.56±0.52	1.19±0.11	0.05±0.004	1.20±0.017	0.81±0.055	0.33±0.018
**Spatc1** (a)	0.30±0.27	0.26±0.045	1.19±0.21	0.81±0.065	0.56±0.066	0.73±0.042
**H2-DMb1** (a)	0.10±0.037	0.58±0.05	0.01±0.016	0.89±0.075	0.72±0.029	0.96±0.023
**Kel** (d)	0.69±0.062	1.04±0.17	0.03±0.002	0.85±0.041	0.61±0.075	0.23±0.034
**Ces2e** (a)	0.69±0.021	9.69±0.61	4.10±0.98	4.60±1.038	0.89±0.058	6.03±1.36

Genes evaluated in RT-PCR experiments in all 6 mouse strains on DRG tissues distinct from those used for microarray experiments. Values express fold-change and are mean + S.E.M. Each group was composed of 6 samples and were analysed in triplicate.

^(a)^ refers to genes that were found significantly changed in Balb-c in microarray experiments;

^(b)^ refers to genes that were found significantly changed in Balb-c and CD1 in microarray experiments;

^(c)^ refers to genes that were found significantly changed in Balb-c and AJ in microarray experiments;

^(d)^ refers to genes that were found significantly changed in AJ in microarray experiments.

Second, we verified if any of the above genes were increased or decreased of at least 1.5-fold compared to control untreated tissues in all 6 strains (Table 6). This led to four genes (Tnnc2, MyH4, CD74 and Siglech) that showed changes in all the strains evaluated. Of the two strains added to this analysis, DBA/2J was the one that showed more profound differences in gene expression changes compared to the other strains. Indeed, 11 out of 15 genes showed similar trends in the five strains, excluding DBA/2J.

## Discussion

Oxaliplatin ((1R,-2R)-1,2-cyclohexanediamine-N,N'][oxalato (2—)-O,O']platinum) is a third generation platinum compound discovered in 1976 and approved by the Food and Drug Administration in 2004 for the treatment of advanced colorectal cancer (http://www.fda.gov/Drugs/DrugSafety/PostmarketDrugSafety). Its therapeutic spectrum was later extended to other malignancies [13]. Subsequently, its efficacy in adjuvant settings (combined with leucovorin and 5-fluorouracil) has also been established leading to an increased number of colorectal cancer patients receiving oxaliplatin. Its clinical use is limited by the onset in patients of 2 clinically distinct forms of peripheral neurotoxicity: a transient and acute syndrome appearing during or shortly after the infusion (affecting up to 95% patients) and a chronic dose-limiting cumulative sensory neuropathy (up to 40–80% of patients) [2].

The risk of developing OIPN is related to several factors including cumulative dose, demographics and co-morbidities. However, the susceptibility to OIPN differs also among patients with similar history and therapeutic regimens suggesting that individual genetic variability can influence the drug neurotoxic response.

To mimic the clinical heterogeneity observed in clinical practice, we employed 6 genetically different mice strains with the aim to evaluate the influence of a different genetic background in determining OIPN phenotypic features. Ten-week-old male mice belonging to Balb-c, C57BL6, AJ, FVB, DBA/2J, CD1 strains were exposed to a well-established chronic schedule of oxaliplatin able to induce a neurotoxic damage without threaten mice general health [1] and tested for the development of peripheral neurotoxicity and neuropathic pain. Moreover, the cumulative dose of oxaliplatin we administered in mice (equivalent to 1080 mg/m2) was in the same range of dosage reported to be neurotoxic in patients [37–39].

The set of outcome measures was selected in order to ascertain the severity of the nervous system damage along the entire sensory pathway (i.e. from the distal nerve endings in the skin up to the spinal dorsal horn wide dynamic range neurons). This was performed with neurophysiological and morphological/morphometric methods as well as with the evaluation of the painful features of peripheral neuropathy at the behavioural level using cold hypersensitivity assessed 24 hours after drug delivery as an indicator of acute neurotoxicity and mechanical threshold as a marker of chronic neurotoxicity. In view of the results previously obtained in Balb-c mice [1] the assessment of nocifensive behaviours was associated with the measurement of WDR spinal dorsal horn electrical activity to evaluate the occurrence of hyperexcitability in response to both noxious (press) and non-noxious (brush) tactile and thermal (acetone) stimuli.

As a general consideration, all mice strains (irrespectively to be inbred or outbred) developed some degree of OIPN, although remarkable differences were observed in its severity and in the specific features, thus supporting the hypothesis that a genetic component might be relevant in this experimental setting and mimicking the clinical situation. Also, it was quite interesting that the parameters measured were not strictly correlated, with strains displaying only selected features over others (Table 4). Outbred strain (CD1) did not show distinct features in comparison to inbred strains.

Overall, mechanical allodynia was significantly present in all 6 strains (albeit to different levels), but this was not true for cold hyperalgesia. This suggests that the molecular mechanisms behind these phenomena are distinct and that the selection of the read-out in the different strains might influence the interpretation of the effects of oxaliplatin administration. These observations, in our opinion, must serve as a warning when devising pre-clinical models to develop new drug treatments.

Balb-c and FVB were the most severely affected strains, with clear damage of all the sensory peripheral nervous system structures investigated associated to acute and chronic nocifensive behaviour. By contrast, C57Bl6 and AJ strains developed a nocifensive behaviour (but only AJ mice showed a robust acute cold allodynia) and different degrees of WDR neurons hyper-excitability with minimal or no evidence of somatic neurotoxicity. DBA/2J and CD1 mice tended to show the opposite results, i.e. only mechanical allodynia in the context of nerve and DRG damage demonstrated with neurophysiological and pathological methods (Table 4).

The differences we described above did not correlate to the quantity of oxaliplatin accumulated in DRGs and sciatic nerves. Moreover, it is very likely that the concentrations of platinum measured after 8 injection of oxaliplatin is abundantly higher than those needed to induce a neurotoxic damage in all the strains considered. Therefore, we do not consider the statistical differences observed between strains in the DRG to be biologically and functionally significant.

This is the first comprehensive pre-clinical study aimed at the comparison of the susceptibility to oxaliplatin peripheral neurotoxicity of different mice strains and, therefore, our results are not thoroughly comparable with literature data. Only data regarding Balb-c mice can be compared with previously reported studies in which this strain was largely employed. As we also confirmed in this study, Renn and collaborators described the development of somatic and painful peripheral neurotoxicity in Balb-c mice treated with a chronic schedule of oxaliplatin [1]. Similar functional alterations of the peripheral nerves were observed also by Wozniak and collaborators in Balb-c mice [40] while exposing animals to a higher treatment regimen close to the maximal tolerated dose (6 mg/Kg twice weekly for 4 weeks). Conversely, our results cannot be compared to numerous other toxicity studies in which oxaliplatin was injected repeatedly for few consecutive days in order to induce nocifensive behaviours [41–43], since it is very unlikely that these schedules can also induce a chronic neurotoxic damage in the peripheral nervous system. However, partial considerations can be done evaluating our results with those of other groups demonstrating that different mice strains can differently respond to other neurotoxic chemotherapy drugs, such as paclitaxel, bortezomib and cisplatin. Smith and colleagues [20] exposed male and female mice belonging to 10 inbred strains to the same short and low dose paclitaxel schedule (0.1 mg/ml, i.p., on 4 alternate days) observing the development of mechanical allodynia with different intensity and severity suggesting a genotype-dependent pattern of responses. DBA/2J and C57BL6 respectively showed a higher and a lower response to paclitaxel treatment. Authors hypothesised, without providing any experimental evidence for their hypothesis, that a common set of genes was responsible for the variability. More recently, Podratz and collaborators found differences in other chemotherapy-induced neurotoxicities between rodent strains using in vitro systems [19]. Cultured DRG from C57BL6 and Balb-c mice exposed to increased concentration of cisplatin (1, 5, 10, 50 μg/ml) were more sensitive than those from DBA/2J and C3H/HeJ. Bortezomib at concentrations of 25, 50, 100, 200 nM induced a biphasic dose response in DBA/2J and C3H/H3J mice. However, C57BL6 DRG and Balb-c DRG were respectively the most and least sensitive to bortezomib [19]. Finally, we observed that the development of mechanical allodynia and the impairment of neurophysiological functions were significantly different across strains (Balb-c, C57BL6, AJ, FVB, DBA/2J, CD1) following chronic cisplatin treatment at the dose of 4 mg/Kg twice weekly for 4 weeks (personal observation).

In addition to our extensive characterization, we also performed a gene microarray analysis of DRG to investigate differences in gene expression. Surprisingly, the observed very high platinum concentration found in DRG did not induce huge changes in gene expression. While some changes were statistically significant, it was evident that only small-to-medium changes occurred. A first observation of our investigation is that some changes occur only in single mouse strains, and this was confirmed by RT-PCR analysis of different samples. Again, this warrants for attention when evaluating single models of OIPN. We also decided to highlight those changes that were evident in all 6 strains in order to gain pilot information for future investigations. The genes that were changed in all 4 strains were unexpected. First, the treated animals showed an enrichment in troponins (Tnnc2) and myosins (MyH4). Tnnc2 and MyH4 showed an increase in all six strains, including DBA/2J and FVB, when tested by RT-PCR but it should be noted that other troponins and myosins also were increased in most strains (Tnnt3v1, Tnnt3v7, TnnI2, MyH8, MyL1, MyBPC2, MyL2, and MyL1v3) Indeed, a literature search did show that troponins ^27,28^ and myosins ^28–31^ are present in DRG ^27,31^ and that others had identified these as up-regulated in this tissue in neuropathic models ^32^. The role of troponins, that our arrays suggest to be expressed in moderate to high levels in DRG, has not been investigated to our knowledge, while a number of reports indicate that myosins are involved in growth cone responses ^30^ and generation of filopodia and lamellipodia ^33^. Whether this is a compensatory effect of the oxaliplatin insult or whether these changes participate in the damage remains to be ascertained.

The 2 down-regulated proteins were equally surprising, as they were a sialic acid binding Ig-like lectin H (Siglech) and CD74. It should be noticed that type-2 histocompatibility complexes (H2-Ab1 and H2-Eb1: MHC) were also significantly decreased in most mouse strains, except for DBA/2J. MHC class II genes have been previously shown to be up-regulated in DRG in models of peripheral nerve injury ^34,35^. It has been suggested that this increase is correlated with invasion of the DRGs by peripheral MHC+ macrophages ^36^. In our model, the fact that MHC class II genes are decreased might instead signify that there is a selective injury to MHC class II+ cells by oxaliplatin although other explanations are possible. CD74 is an extracellular surface receptor for macrophage migration inhibitory factor and therefore it is not surprising that it correlates with a decrease in MHC-type II genes. A different involvement of the macrophage component in OIPN compared to other peripheral neuropathies would obviously be of great importance but needs confirmation with other approaches. Again, it remains to be ascertained whether these would be compensatory mechanisms or a root cause of the damage.

The above data altogether suggest that the four identified genes should be the focus of further investigation in the context of OIPN. Particular attention should be devoted to CD74 and Siglech, which were both formally identified via stringent adjusted statistical significance in two strains and by supervised analysis in all six strains. Furthermore, the data also suggest novel study designs that should overcome in the future some of the limits of our approach. First, it is apparent from the nature of the identified genes that the cell origin of these changes might be non-neuronal. While this opens new scenarios on how OIPN might be triggered (for example, with an involvement of satellite cells), it also sets a confounding factor, given that satellite cells, infiltrating cells and connective tissue might be dominant over sensory neurons. Future experiments should undertake microarray experiments on sorted isolated cells (both neuronal and non-neuronal) to unravel the origin of the oxaliplatin-induced changes and to identify neuronal changes as well. Second, the microarray experiments were not performed on the same animals that underwent the behavioural/electrophysiological/histological tests and therefore we cannot reconcile the single genotype to the single phenotype. Given the intra-strain variability that we observed future behavioural and microarray experiments should be performed on the same animals, as this would allow to compare highly sensitive and less sensitive mice on parameters that are relevant to human disorder (e.g. nerve conduction).

## Conclusions

In conclusion, this work demonstrated that different mice strains, exposed to the same oxaliplatin treatment, do have different neurotoxic phenotypes. This is of particular interest in choosing the most appropriate strain to be employed, based on the selected read-out parameter in the neurotoxicity study. Similar conclusions were obtained when evaluating gene expression changes induced by oxaliplatin in whole DRGs. Moreover, the genetic analysis demonstrated that oxaliplatin determines small-to-medium gene expression changes. Notwithstanding the above considerations, the expression of CD74 and Siglech was reduced in all strains considered, suggesting that common features exist.

Our approach, and the genes highlighted, also suggest that alongside investigating the role of these genes, future work should be directed at (i) investigating gene expression changes in isolated DRG populations (to dissect the neuronal and non-neuronal cells) and (ii) performing microarray analysis on the same animals that undergo phenotypic evaluation, to better correlate gene expression changes to phenotypic characteristics, bypassing intra-strain variability.

## Supporting information

S1 File Rank product method. Non-parametric statistical test based on ranks of FC (or logFC) expression values.
(DOCX)

S1 Fig Venn diagrams of gene changes observed in the four mouse strains tested in microarray experiments. (A, B) Venn diagram of the genes that displayed significant changes after statistical adjustment for multiple comparisons. C57BL6 results are not depicted as no significant changes were observed; (C, D) Venn diagram of gene changes emerged in the supervised analysis in the four mouse strains tested in the microarray experiments, independently of statistical significance.
(TIF)

S1 Table List of primers used in real time quantitative PCR. (DOCX)

S2 Table Spinal cord electrophysiology. The table shows the electrical activity of the spinal dorsal horn wide dynamic range neurons expressed as a mean ± SD of the number of spike /second measured in naive and oxaliplatin-treated animals of each strain. The change (%) of the neuronal electrical activity of oxaliplatin-treated versus respective naive animals is also reported. The electrical activity of CD1 mice was not recordable for technical reasons.
(DOCX)

S3 Table Platinum concentration. The table shows the mean ± SD of platinum concentration (μg platinum/μg of tissue or /ml of plasma) measured in plasma, DRG and sciatic nerves of oxaliplatin-treated animals of each strain.
(DOCX)

S4 Table Upregulated and downregulated genes based on LogFC value. (DOCX)
