# Supplementary material for: Susceptibility of different mouse strains to oxaliplatin peripheral neurotoxicity: Phenotypic and genotypic insights
Source: PLoS One. 2017 Oct 11;12(10):e0186250. doi: 10.1371/journal.pone.0186250 (PMC5636145; doi:10.1371/journal.pone.0186250)
Supplement: S1 File — Non-parametric statistical test based on ranks of FC (or logFC) expression values. (DOCX) [file pone.0186250.s001.docx]

**S1 File**

**Rank product test**

This algorithm, firstly reported by Breitling et al. (2004), is a non-parametric statistical test based on ranks of FC (or logFC) expression values. In particular, it allows detecting genes that are consistently changed within a set of microarray experiments, namely finding all genes that are significantly up- or down-regulated across the different samples and strains in the whole data set. In general, given *n* genes and *k* microarray experiments (or samples), the rank product *RP* is computed for each gene *g* via geometric mean as$RP\left( g \right)=\left( \prod_{i=1}^{k} r_{g,i} \right)^{1/k}$, where $r_{g,i}$ is the rank of gene *g* in the list of genes in the *i*-th experiment sorted by increasing or decreasing FC. A permutation-based estimation is then used as null model to determine how likely a given *RP* value (or better) is observed in a random experiment. This provides a reliable way to determine the significance level for each gene and allows for the control of the false discovery rate (FDR) or familywise error rate (FWER) for multiple comparisons. For a more detailed description, see Breitling et al. (2004) and Heskes et al. (2014). In our case, the algorithm was applied to the whole dataset controlling the FDR at 5% level. All the genes selected on the basis of the more simple (yet arbitrary) logFC criterion (see main text, Table 4) find a match in the most high-ranked entries of both the up- and down-regulated gene lists returned by RP procedure, thus statistically validating and extending at the same time the former logFC-based gene selection.

Breitling, R., Armengaud, P., Amtmann, A. & Herzyk, P. Rank products: a simple, yet powerful, new method to detect differentially regulated genes in replicated microarray experiments. *FEBS Lett* **573**, 83-92, doi:10.1016/j.febslet.2004.07.055 (2004).

Heskes, T., Eisinga, R. & Breitling, R. A fast algorithm for determining bounds and accurate approximate p-values of the rank product statistic for replicate experiments*. BMC Bioinformatics* **15**, 367, doi:10.1186/s12859-014-0367-1 (2014).
