# Supplementary figures and images for: Susceptibility of different mouse strains to oxaliplatin peripheral neurotoxicity: Phenotypic and genotypic insights
Source: PLoS One. 2017 Oct 11;12(10):e0186250. doi: 10.1371/journal.pone.0186250 (PMC5636145; doi:10.1371/journal.pone.0186250)

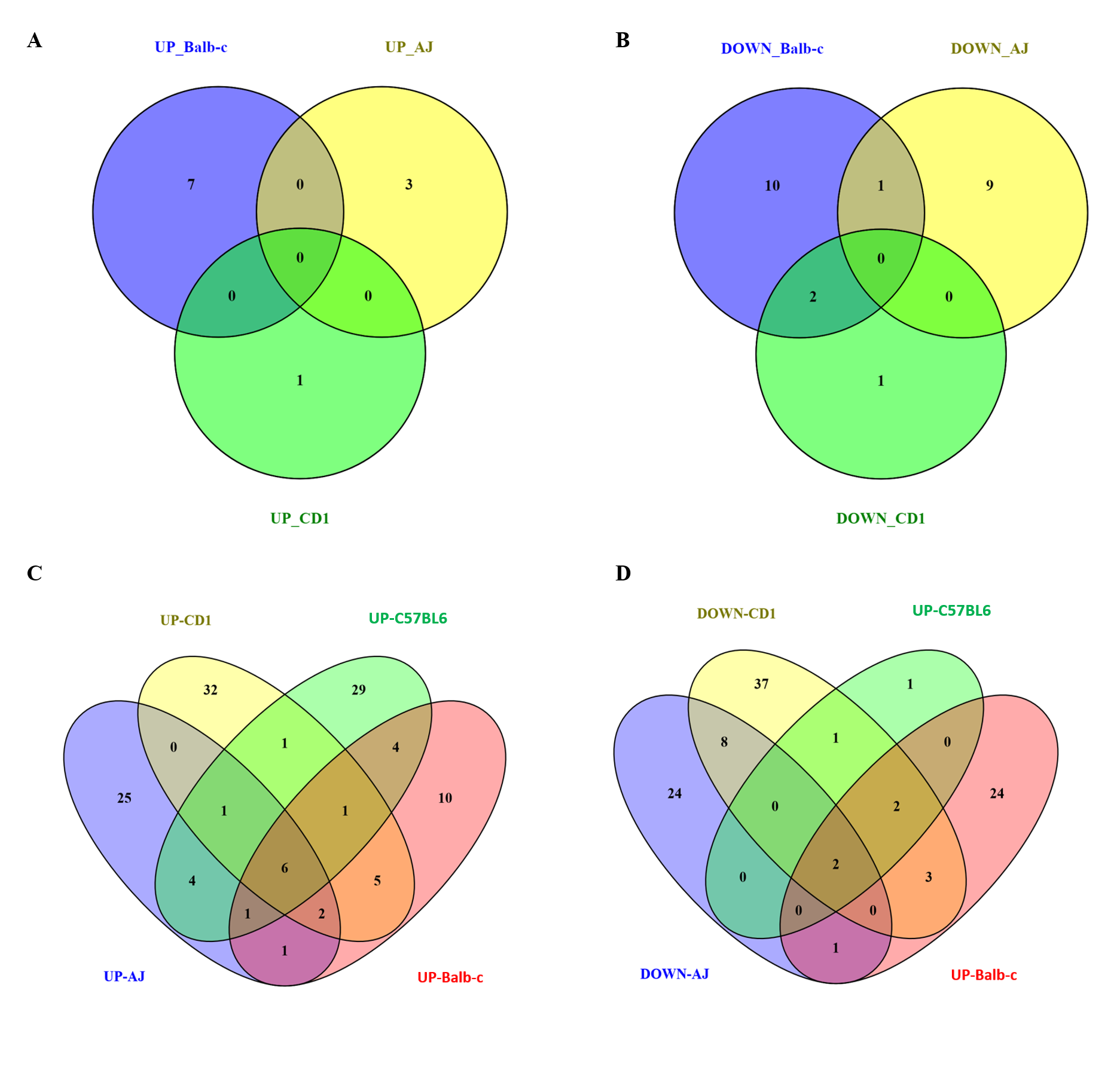

Supplement: S1 Fig — (A, B) Venn diagram of the genes that displayed significant changes after statistical adjustment for multiple comparisons. C57BL6 results are not depicted as no significant changes were observed; (C, D) Venn diagram of gene changes emerged in the supervised analysis in the four mouse strains tested in the microarray experiments, independently of statistical significance. (TIF) [file pone.0186250.s002.tif]
