# Supplementary material for: Susceptibility of different mouse strains to oxaliplatin peripheral neurotoxicity: Phenotypic and genotypic insights
Source: PLoS One. 2017 Oct 11;12(10):e0186250. doi: 10.1371/journal.pone.0186250 (PMC5636145; doi:10.1371/journal.pone.0186250)
Supplement: S1 Table — (DOCX) [file pone.0186250.s003.docx]

**S1 Table. List of primers used in real time quantitative PCR.**

| **Gene Symbol** | **Primers** |
| --- | --- |
| **Tnnc2** | FW 5’- CAGTCGCCAGCAACCATG-3’, |
|  | RV 5’- CCTAGCATCCTCATCACGGT-3’ |
| **Myh8** | FW 5’-CCACCCCACATCTTCTCCAT-3’ |
|  | RV 5’- CGCTTCGTGTTCACAGTCTT-3’ |
| **Tnnt3 v1** | FW 5’-GTCCAAGAAGATGCTGTCGC-3’ |
|  | RV 5’-TCTACTTTCTCCCCTTCCGG-3’ |
| **Myh4** | FW 5’-GCCCAGTGGAGGACGAAATA-3’ |
|  | RV 5’-GCGCATTTGGAATTCACAGC-3’ |
| **Ckm** | FW 5’-CTTCCTGTTTGACAAGCCCG-3’ |
|  | RV 5’-CGTTCACCCACACAAGGAAG-3’ |
| **Mb v2** | FW 5’-TGGTGCTGAATGTCTGGGG-3’ |
|  | RV 5’-TATCCAGGGTCTCAGGGTGA-3’ |
| **Myl1** | FW 5’-GAAAGACGTGAAGAAGCCCG-3’ |
|  | RV 5’-ATGCCTCCTTGAAGTCCTCC-3’ |
| **Mybpc2** | FW 5’-AGAGCCTCGAGAGCTTCAAG-3’ |
|  | RV 5’-CGCCTGCATCTTCTGACTTC-3’ |
| **Acta1** | FW 5’-GGGCCAGAGTCAGAGCAG-3’ |
|  | RV 5’-GCCAGAGCCGTTGTCACA-3’ |
| **Tnnt3 v7** | FW 5’-AACAAGTTGAGGAGGAGGCC-3’ |
|  | RV 5’-TCTACTTTCTCCCCTTCCGG-3’ |
| **Pgam** | FW 5’-TGGAATGAGATCGCACC-3' |
|  | RV 5’-CAGGTTCTGGTCCAGCTCAT-3' |
| **Cox6a2** | FW 5’-CTAAAGGTCCTGAGCCGGAG-3' |
|  | RV 5’-ATACGGGATGAACTCTGGGC-3' |
| **Myl2** | FW 5’-GGACACATTTGCTGCCCTAG-3’ |
|  | RV 5’-TCCCCAAACATCGTGAGGAA-3’ |
| **Tcap** | FW 5’-CTGCCAAGTGTCTGAGGAGA-3' |
|  | RV 5’-TCTGTGTATCCTCCTCGTGC-3' |
| **Tnni2** | FW 5’- AGGCTCCATGTCTGAAGTGC-3’ |
|  | RV 5’- GCTTCTGGTTCATGTCTTCCA-3’ |
| **Myl1 v3** | FW 5'-TCCACTGTGACAAGCTGCAT-3' |
|  | RV 5'-CCTCCCTGGTCCTTGTTGTT-3' |
| **Siglech** | FW 5'-TTCACAGAACTCCACAGCCC-3' |
|  | RV 5'-TTTGATAGCCGCTTCTCCCA-3' |
| **H2-Ab1** | FW 5'-CAACCACCACAACACTCTGG-3' |
|  | RV 5'-ATCTCCAGCATGACCAGGAC-3' |
| **CD74** | FW 5’-AAGAACGTTACCAAGTACGGC-3’ |
|  | RV 5’-CTTCCAGTTCACGCCATCCA-3’ |
| **H2-Eb1** | FW 5’-CTCCTGGTCTGCTCTGTGAG-3’ |
|  | RV 5’-CCACTCTGAGGAACCGTCTC-3’ |
| **Ctse** | FW 5’- GCCAGACCTTTGTGAATGCA-3’ |
|  | RV 5’- TGTCAAACACTGGGGTCACT-3’ |
| **Pttg1** | FW 5’- GCAAACCCCTCCAACCAAAA-3’ |
|  | RV 5’- TAGGCATCATCAGGAGCAGG-3’ |
| **Gm684** | FW 5’- GAACCAACGTCTTCCACACC-3’ |
|  | RV 5’- CGGGAGTGTGTCTGTCTGAA-3’ |

| **Gene Symbol** | **Primers** |
| --- | --- |
| **Rhd** | FW 5’- GATTTGCCGACGAGAAGGTC-3’ |
|  | RV 5’- CACTCTCCTGGGCAGAGATC-3’ |
| **Spatc1** | FW 5’-AAGTCCTTCCTCAGCCCTTC-3’ |
|  | RV 5’-AGCAGTGTTTTGTCAGCCAG-3’ |
| **H2-DMb1** | FW 5’- GCTACGTCTGGGGCTTCTAT-3’ |
|  | RV 5’- TGAGCCGTCTTCTCCTTGTT-3’ |
| **Kel** | FW 5’- GATCTTGGGGCTGGTGGATA-3’ |
|  | RV 5’- CCGTCTGTTCCACACACTTC-3’ |
| **Ces2e** | FW 5’-CTTCGTTGGGTCCAGCAAAA-3’  RV 5’-CCACCTGCTGACTCTCCAAA-3’ |
