# Supplementary material for: Susceptibility of different mouse strains to oxaliplatin peripheral neurotoxicity: Phenotypic and genotypic insights
Source: PLoS One. 2017 Oct 11;12(10):e0186250. doi: 10.1371/journal.pone.0186250 (PMC5636145; doi:10.1371/journal.pone.0186250)
Supplement: S2 Table — The table shows the electrical activity of the spinal dorsal horn wide dynamic range neurons expressed as a mean ± SD of the number of spike /second measured in naive and oxaliplatin-treated animals of each strain. The change (%) of the neuronal electrical activity of oxaliplatin-treated versus respective naive animals is also reported. The electrical activity of CD1 mice was not recordable for technical reasons. (DOCX) [file pone.0186250.s004.docx]

**S2 TABLE. Spinal cord electrophysiology.**

| **NEURONAL ELECTRICAL ACTIVITY, BRUSH (spike/sec)** | | |  |  |
| --- | --- | --- | --- | --- |
| **NAIVE** | **OHP** | **CHANGE vs NAIVE (%)** |  |  |
| **Balb-c** | | 92.0 ± 10.27 | 129.1 ± 12.42 (#) | 40.2 |
| **C57BL6** | | 125.9 ± 15.46 | 182.1 ± 19.92 (#) | 44.4 |
| **AJ** | | 38.6 ± 6.24 | 138.3 ± 13.97 (***) | 238.0 |
| **FVB** | | 103.7 ± 8.51 | 106.5 ± 8.74 | 2.9 |
| **DBA/2J** | | 73.5 ± 13.62 | 80.8 ± 8.22 | 9.8 |
| **CD1** | | ND | ND |  |
| **NEURONAL ELECTRICAL ACTIVITY, PRESS (spike/sec)** | | |  |  |
| **NAIVE** | **OHP** | **CHANGE vs NAIVE (%)** |  |  |
| **Balb-c** | | 70.2 ± 10.48 | 142.7 ± 26.23 (#) | 102.0 |
| **C57BL6** | | 106.1 ± 16.71 | 115.7 ± 11.08 | 8.5 |
| **AJ** | | 35.8 ± 7.62 | 79.2 ± 9.08 (*) | 119.0 |
| **FVB** | | 81.3 ± 5.75 | 109.8 ± 16.73 | 34.6 |
| **DBA/2J** | | 75.5 ± 11.37 | 64.6 ± 12.79 | -14.0 |
| **CD1** | | ND | ND |  |
| **NEURONAL ELECTRICAL ACTIVITY, ACETONE (spike/sec)** | | |  |  |
| **NAIVE** | **OHP** | **CHANGE vs NAIVE (%)** |  |  |
| **Balb-c** | 6.7 ± 1.51 | 12.9 ± 2.12 (#) | 93.4 |  |
| **C57BL6** | 5.4 ± 1.00 | 11.4 ± 1.38 (*) | 111.0 |  |
| **AJ** | 7.1± 1.76 | 28.4 ± 6.94 (**) | 297.0 |  |
| **FVB** | 2.2 ± 0.38 | 5.2 ± 1.40 (#) | 132.0 |  |
| **DBA/2J** | 3.9 ± 1.15 | 2.3 ± 1.09 | 40.3 |  |
| **CD1** | ND | ND |  |  |

**The table shows the electrical activity of wide dynamic range neurons of the spinal dorsal horn ~~of the spinal cord~~ expressed as a mean ± SD of the number of spike/second measured in naive and oxaliplatin-treated animals of each strain. The change (%) of the neuronal electrical activity of oxaliplatin-treated versus respective naive animals is also reported. The electrical activity of CD1 mice was not recordable for technical reasons.**

*****p<0.0001; **p<0.001; *p<0.01; #p<0.05 vs NAIVE; Student t test**
