# Supplementary material for: Susceptibility of different mouse strains to oxaliplatin peripheral neurotoxicity: Phenotypic and genotypic insights
Source: PLoS One. 2017 Oct 11;12(10):e0186250. doi: 10.1371/journal.pone.0186250 (PMC5636145; doi:10.1371/journal.pone.0186250)
Supplement: S3 Table — The table shows the mean ± SD of platinum concentration (μg platinum/μg of tissue or /ml of plasma) measured in plasma, DRG and sciatic nerves of oxaliplatin-treated animals of each strain. (DOCX) [file pone.0186250.s005.docx]

**S3 TABLE. Platinum concentration.**

|  | **[PLATINUM] (µg/g tissue or µg/ml plasma)** | | |
| --- | --- | --- | --- |
|  | **PLASMA** | **DRG** | **SCIATIC NERVE** |
| **Balb-c** | 0.18±0.03 | 14.03±0.55 # | 5.64±2.72 |
| **C57BL6** | 0.21±0.05 | 10.72±1.73 ** | 5.12±1.16 |
| **AJ** | 0.12±0.01 | 20.43±2.26 | 9.5±3.58 |
| **FVB** | 0.16±0.03 | 12.07±2.12 ** | 7.42±3.20 |
| **DBA/2J** | 0.19±0.05 | 10.80±0.99 ** | 4.03±0.41 |
| **CD1** | 0.078±0.03 | 15.56±4.15 | 7.73±5.78 |

**The table shows the mean ± SD of platinum concentration (µg platinum/g of tissue or /ml of plasma) measured in plasma, DRG and sciatic nerves of oxaliplatin-treated animals of each strain.**

****p<0.001, #p<0.05 vs AJ; One Way ANOVA, Tukey post test**
