# Supplementary material for: Susceptibility of different mouse strains to oxaliplatin peripheral neurotoxicity: Phenotypic and genotypic insights
Source: PLoS One. 2017 Oct 11;12(10):e0186250. doi: 10.1371/journal.pone.0186250 (PMC5636145; doi:10.1371/journal.pone.0186250)
Supplement: S4 Table — (DOCX) [file pone.0186250.s006.docx]

**S4 TABLE. Upregulated and downregulated genes based on LogFC value.**

**Up-regulated genes**

**Down-regulated Genes**
